# Inhibitory effects of mycosubtilin on proliferation of colon cancer SW480 cells

**DOI:** 10.1371/journal.pone.0348056

**Published:** 2026-05-20

**Authors:** Jun Yang, Qiong Wu, Qian Zhou, Li Yin, Huixin Zhao, Jinyu Li

**Affiliations:** 1 Xinjiang Key Laboratory of Special Species Conservation and Regulatory Biology, College of Life Science, Xinjiang Normal University, Urumqi, China; 2 Beijing Key Laboratory of Gene Resource and Molecular Development, College of Life Sciences, Beijing Normal University, Beijing, China; Texas A&M University, UNITED STATES OF AMERICA

## Abstract

The development of novel and effective natural anti-cancer drugs represents a significant focus in contemporary biomedical research. Mycosubtilin (Myco), a cyclic lipopeptide produced by *Bacillus subtilis*, has not previously been investigated for its potential anti-colorectal cancer activity. This study employed a comprehensive approach combining network pharmacology, molecular docking, cellular analysis, flow cytometry single-color staining, and western blot analysis to preliminarily investigate the potential molecular mechanisms underlying Myco’s anti-CRC activity. Findings revealed that Myco significantly outperformed 5-Fluorouracil (5-FU) in inhibiting SW480 cell proliferation. Myco effectively triggered apoptosis. This was supported by the typical apoptotic morphological features, including nuclear condensation and fragmentation. Concurrently, Myco treatment downregulated the expression of the anti-apoptotic protein, Bcl-2. These findings suggest that Myco has potential application value in inhibiting the proliferation of human colorectal cancer SW480 cells.

## Introduction

Colorectal cancer (CRC) is currently one of the most prevalent malignant tumors, with its incidence having significantly increased over the past five decades. In 2023, approximately 153,020 individuals are expected to be diagnosed with CRC, and 52,550 will succumb to the disease [1]. CRC has now become the third most common malignant tumor worldwide. Despite advancements in treatment, the management of advanced and recurrent CRC remains inadequate, resulting in low cure rates. Furthermore, chemotherapy agents can induce severe toxic side effects and compromise the patient’s immune system, while patients are gradually developing increased resistance to these drugs [2]. To address these challenges, there is an urgent need for the development of novel anti-colorectal cancer therapeutics.

The screening and evaluation of anticancer drugs primarily involve studies on their capacity to inhibit tumor angiogenesis, suppress cell invasion/migration, regulate cell cycle progression, induce apoptosis, and trigger autophagy. Among these mechanisms, key proteins in the intrinsic apoptotic pathway such as Bax and Bcl-2 and cell cycle regulators like CDK6 and Cyclin D1 are increasingly used as clinical therapeutic targets [3–5]. An emerging strategy, a network pharmacology approach using computer science and systems biology to predict drug active ingredients and disease targets, has the role of constructing biological networks connecting drugs, active ingredients [6,7], targets and diseases revealing the interrelationships between these elements, and has been widely used in drug active ingredient screening and mechanism exploration.

In recent years, the discovery and screening of lipopeptides have opened new frontiers in cancer therapy. *Bacillus subtilis* produces a diverse array of biologically active lipopeptides, including iturins, surfactins, and fengycins [8]. These compounds exhibit notable advantages owing to their biodegradability and high safety profiles. Emerging studies have highlighted their therapeutic potential in oncology [9,10]. Among them, the iturin family comprises Iturin A, Iturin D, Bacillomycin and Mycosubtilin (Myco). Although these molecules share similar structural backbones, their biological activities diverge significantly because of variations in peptide chain structures and fatty acid carbon chain lengths. Myco, in particular, stands out of the most potent antifungal lipopeptides [11]. Structural modifications have been shown to alter toxicity and mechanisms of action across different cancer cell types, with surfactin, iturin, and bacillomycin D exhibiting inhibitory effects against melanoma, leukemia, and lung adenocarcinoma cells in previous studies [12–14]. These findings suggest that lipopeptides may exhibit differential cytotoxic effects among various cell types, possibly reflecting differences in cellular sensitivity, and therefore have the potential to become promising antitumor agents. Studies have shown that lipopeptides from *Bacillus subtilis* exhibit significant inhibitory effects on various tumor cells through multiple mechanisms and targets [15,16]. Compared to other structures, Myco has altered amino acid positions and conformations [17]; thus, a deeper understanding of its impact on cancer cell apoptosis is crucial for the clinical promotion of *Bacillus subtilis* lipopeptides and the expansion of their broad-spectrum cancer treatment capabilities. As a naturally derived lipopeptide, Myco has attracted attention because of its biodegradability and biological activity; however, its antitumor potential remains insufficiently explored. While research on Myco’s antitumor activity remains limited, preliminary evidence shows that mixtures containing Myco exhibit significant inhibitory effects on hepatocellular carcinoma [18] and cervical cancer [19] cell lines, and immunomodulatory effects [20]. However, the specific role of Myco in CRC remains unclear. This study aimed to determine whether Myco inhibits CRC cell growth and elucidate its underlying molecular mechanisms, providing a theoretical foundation for Myco as a potential therapeutic agent in CRC.

## Materials and methods

### Materials

RNA Extraction Kit, GoTaqqPCRMasterMix Kit purchased from GoTaq® qPCR Master MixPromega (Beijing) Biotechnology Co. TUNEL Apoptosis Detection Kit was purchased from Next Sense Biotechnology (Shanghai) Co. Human colon cancer cell line SW480 was purchased from Sunshine Biotechnology (Shanghai) Co. The chemicals used in this study were purchased from Beijing Solepol Technology Co. Including Glutaraldehyde, Dimethyl sulfoxide (DMSO), 3-(4,5-Dimethylthiazol-2-yl)-2,5-diphenyltetrazolium bromide (MTT), Hanks’ Balanced Salt Solution (HBSS), Iscove’s Modified Dulbecco’s Medium (IMDM), Polyvinylidene Fluoride (PVDF), FBS, PBS, BEP medium, Pen-Strep, Sodium Chloride, Potassium Chloride.

*Bacillus subtilis* BS-Z15 and the human colon cancer cell line SW480 were preserved through laboratory propagation. Myco was prepared following the method described by Lin et al. and subsequently purified using a semi-preparative high-performance liquid chromatography system (C18, 5 μm, 250–10 mm, Hypersil GOLDTM, CA) to obtain Myco [21].The purity of the obtained Myco was determined to be 99% (S1 Fig).

### Screening of Myco’s action targets and molecular docking in CRC

Pharmacological targets of Myco were predicted using databases such as BATMAN-TCM and bSDTNBI, identifying a total of 709 targets. Concurrently, 5,473 therapeutic targets associated with CRC were retrieved from the DisGeNET database. Subsequently, 25 key potential target proteins were screened using the STRING database and Cytoscape software. All 25 potential targets were CRC-related therapeutic targets, and a network pathway diagram was constructed. The protein targets, accession numbers, and PDB structures used for molecular docking analysis are listed in S1 Table. Furthermore, bioinformatics analysis was performed on these 25 potential targets using the DAVID bioinformatics software. This included Gene Ontology (GO) analysis and KEGG pathway enrichment analysis.

To validate the accuracy of the network pharmacology predictions, docking was performed between Myco and the key Bcl-2 target. Protein and ligand processing was conducted using pymol software. AutoDockTools-1.5.6 was employed to convert the processed proteins and ligands into compatible formats. Finally, molecular docking analysis was conducted using the AutoDockVina program, visualized with Pymol, and analyzed in two dimensions using Ligplot.

### Cell proliferation capacity assay

Upon reaching 80% confluency, SW480 cells were digested using a 0.25% trypsin-EDTA, mixed with IMDM, and subsequently counted. The cells were then seeded into 96-well plates at a density of 6000 cells per well and cultured overnight. Various concentrations of Myco (1, 2, 4, 8 and 16 μg/mL) and 5-Fluorouracil (5-FU 1, 3, 9, 27, 81 and 243 μg/mL) were added to the wells designated for the experimental group, with six replicate wells established for each concentration. An equal volume of medium was added to the control group, while the zeroing group was maintained without cells. Following a 72-h incubation period, the medium was discarded, and the wells were washed with HBSS. Subsequently, 100 μL of MTT (0.5 mg/mL) was added to each well and incubated for 4 hours. After the removal of the MTT solution, 150 μL of DMSO was added to each well, and the cells were incubated at 37 °C for 10 min. The absorbance was then measured at 570 nm using an enzyme marker.

### Cell morphology observation

In this study, SW480 cells at passage 10 in the logarithmic growth phase were used. Cells were routinely cultured in a humidified incubator at 37 °C with 5% CO₂. Given that the IC₅₀ value of Myco against SW480 cells was 6.15 μg/mL, 4 μg/mL and 8 μg/mL were selected to observe cell morphology. After seeding, SW480 cells were treated with Myco at final concentrations of 4 μg/mL and 8 μg/mL, with three replicate wells per group. Following 48 h of drug treatment, the culture medium was discarded, and cells were washed with PBS, then fixed overnight at 4 °C with 2.5% glutaraldehyde. After being washed three times with PBS, cells were dehydrated in a graded ethanol series (50%, 70%, 80%, 90%, 95%, 100%) for 15 min each. After natural drying for 30 min, the samples were placed in a desiccator containing blue silica gel for at least 24 h. The dried cell samples were mounted on aluminum stubs with conductive carbon tape, and coated with an 8–10 nm gold‑palladium alloy (Au/Pd, 6:4) using an ion sputter coater. Sputtering was performed under high vacuum (10 ⁻ ³–10 ⁻ ⁴ Pa) with high‑purity argon as the working gas at a pressure of 5 Pa. After pre‑sputtering for 60 s to remove impurities from the target, formal sputtering was conducted at 20 mA for 90 s with continuous rotation of the sample stage to ensure uniform coating. Cell ultrastructure was observed under a scanning electron microscope (JEOL it300).

For additional morphological observation, SW480 cells in the logarithmic growth phase were seeded in 10 cm culture dishes. After cell adherence, cells were treated with Myco at 4, 8, and 16 μg/mL, respectively. After incubation for 48 h, cell morphological changes were observed using a long‑term label‑free live cell imaging system.

### Apoptosis detection

SW480 cells were seeded in culture dishes at a density of 1 × 10^4^ cells per milliliter and incubated for 12 h to promote adherence. With the exception of the fluorescence compensation group, Myco was added to the experimental group to a final concentration of 8 μg/mL, while 5-FU was added to the positive control group to a final concentration of 8 μg/mL. Three replicate wells were set up for each group. Notably, the negative control group received only IMDM medium with an equivalent volume of DMSO. After 48 h of culture, SW480 cells were collected and resuspended to a concentration of 1 × 10^6^ cells/mL in a binding buffer. Next, 5 μL of AnnexinV-FITC was added, and the mixture was incubated for 15 min at room temperature, protected from light. Following incubation, 5 μL of propidium iodide solution was added, and the cells were resuspended by adding 200 μL of binding buffer to the reaction tubes. The samples were then analyzed using flow cytometry.

### Western blotting

SW480 cells in the logarithmic growth phase were collected, and Myco was added to the experimental group to a final concentration of 8 μg/mL. A control group was also set up. After 48 h of culture, the cells were harvested and lysed using cell lysis buffer. Protein concentration was then quantified using the Bradford method and measured with an enzyme marker. Loading buffer was added to the protein samples, which were then heated at 100 °C for 5 min to achieve complete denaturation and subsequently cooled to room temperature. The protein samples were resolved by electrophoresis through a 12% SDS-PAGE gel. PVDF membranes were pre-activated in anhydrous methanol for 10s, and the protein samples were transferred onto the membranes at a constant current of 200 mA for 1 h. Next, the PVDF membrane was treated in a containment solution at 37 °C for 1 h. The antibodies were diluted in a 5% skimmed milk solution, with the primary and secondary antibodies diluted to concentrations ranging from 1:1000–1:3000, respectively. The PVDF membrane was incubated in the primary antibody solution at 4 °C for 12 h, followed by four washes with TBST, each for 5 min. The membrane was then transferred to the secondary antibody solution and incubated at room temperature for 1 h, followed by another four washes with TBST, each for 5 min. Finally, the PVDF membrane was placed face up, and the color development mixture was added dropwise for detection.

### Detection of nuclear morphology in apoptosis and DNA damage in cells

SW480 cells were seeded at a density of 10,000 cells per well in 6-well plates pre-coated with coverslips and cultured for 12 h. The experimental group was treated with Myco to achieve a final concentration of 8 μg/mL, while the control group received IMDM containing an equivalent volume of DMSO. Following a 48h culture period, Hoechst staining and the TUNEL apoptosis assay were performed in accordance with the manufacturer’s instructions. Fluorescence was observed using an inverted fluorescence microscope.

### Reactive oxygen species (ROS) detection

SW480 cells were seeded into confocal dishes at a density of 10,000 cells/mL and cultured for 12 h. The experimental group was treated with Myco at a final concentration of 8 µg/mL, while the positive control group was treated with Rosup (ROS Up-Regulator) for 25 min. After 48 h of culture, the medium was discarded, and the cells were incubated with 10 µM of the ROS fluorescent probe (DCFH-DA) at 37 °C for 20 min. Following three washes with serum-free medium, 2 mL of serum-free medium was added. The results were visualized using laser confocal microscopy.

### Detection of intracytoplasmic calcium signaling

SW480 cells were seeded at a density of 10,000 cells/mL into laser confocal dishes and cultured for 12 h. The culture medium was discarded, cells were rinsed with HBSS, and imaging buffer containing the calcium fluorescent probe Fura-2 AM (5 μM) was added. Cells were incubated in the dark at 37 °C for 30 min to allow dye loading, then washed twice with fresh imaging buffer to remove extracellular dye.

Calcium imaging was performed using an inverted fluorescence microscope equipped with a photometry and imaging system. Fura-2 fluorescence was excited alternately at 340 nm and 380 nm using a high-speed wavelength switcher, and emission was collected at 510 nm. After a brief baseline recording, intracellular calcium stores were depleted by treatment with ionomycin. The ionomycin solution was then removed, and Myco was added to the experimental group at final concentrations of 6, 8, and 10 μg/mL. Changes in calcium signaling were monitored by calculating the F340/F380 ratio over time. Data were analyzed using the imaging system software.

### Statistical analysis

Statistical analysis was performed using GraphPad Prism 9.0. Differences were analyzed using the unpaired twosample t test. The data are presented as the means ± standard errors of the means (SEM) or standard deviations (SD). A p value less than 0.05 was considered statistically significant.

## Results

### Prediction of Myco anti-CRC targets and molecular docking of key targets

**Fig 1:**
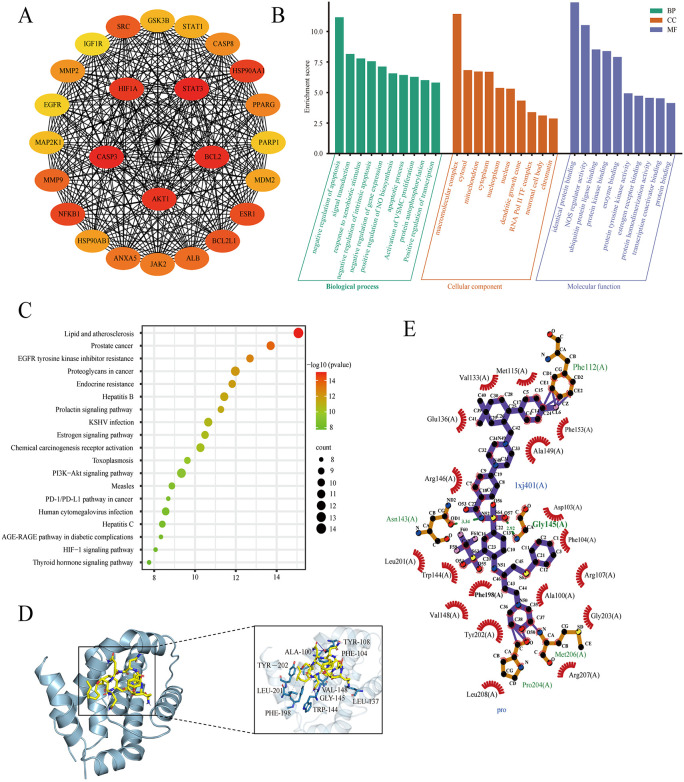
Network pharmacology of Myco’s anti-CRC mechanism and molecular docking analysis of target proteins. **(A)** Protein network pathway map of potential targets, with darker node colors indicating a higher degree of relevance. **(B)** GO functional annotation of Myco anti-CRC potential targets, including biological process (BP), cellular component (CC), and molecular function (MF). **(C)** KEGG enrichment analysis of potential signaling pathways. The top 20 signaling pathways with lower p-values were visualized. **(D)** Three-dimensional view of Myco docking with Bcl-2 molecules. **(E)** Two-dimensional diagram of Myco docking with Bcl-2 molecules.

A comprehensive analysis of 25 pharmacological targets associated with Myco was conducted using database search, resulting in the identification of 5473 genes related to CRC. All 25 pharmacological targets were found to overlap with the disease targets of CRC. The STRING database was used to construct a protein-protein interaction (PPI) network diagram, illustrating the common targets between drugs and the disease (Fig 1A). To thoroughly investigate the anti-CRC mechanisms of Myco, GO gene enrichment analysis, and KEGG pathway analysis were performed on the 25 drug-disease targets. GO enrichment analysis revealed that the effects of Myco on rectal cancer encompass a range of biological processes, including negative regulation of apoptotic process, signal transduction, and response to xenobiotic stimulus. In addition, Myco influences various cellular components, such as the macromolecular complex, cytosol, and mitochondria. The analysis also indicated involvement in molecular functions, such as identical protein binding, nitric-oxide synthase regulator activity, and ubiquitin protein ligase binding (Fig 1B). KEGG enrichment analysis showed that the key targets of Myco in antagonizing rectal cancer were mainly associated with pathways in cancer, lipid and atherosclerosis, and prostate cancer. Notably, the PI3K/AKT signaling pathway, which is closely related to the development of rectal cancer, was identified as a significant pathway (Fig 1C).

To validate these predictions, we performed molecular docking of Myco with the Bcl-2 protein. The results showed that Myco exhibits binding affinity to the key target protein Bcl-2, with a binding energy of −5.9 kcal mol^–1^. Myco can interact with Bcl-2 by forming hydrogen bonds through 108A, 144A, and 145A (Figs 1D-E).

### Myco affects the cell structure and morphology of SW480 cells and inhibits cell proliferation

**Fig 2:**
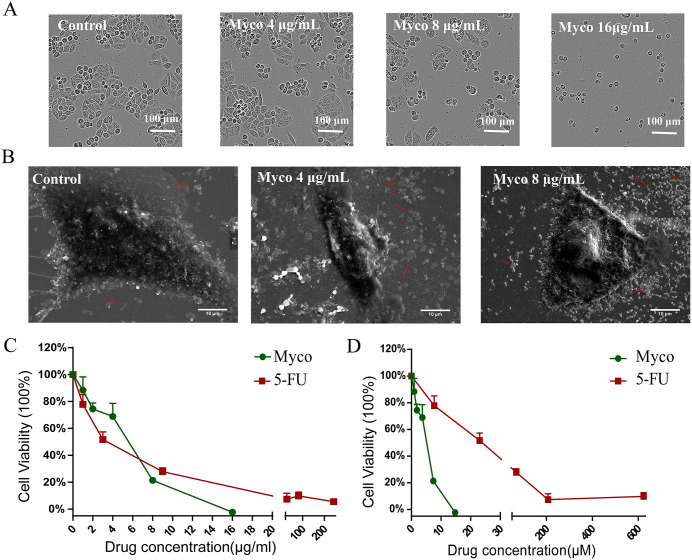
Effects of Myco on the morphology and proliferation of SW480 cells. **(A)** Microscopic view of SW480 cell morphology, scale bar represents 100 μm. **(B)** Scanning electron microscopy image of SW480 cells, Magnification: 2000 × , scale bar represents 10 μm. **(C)** Changes in SW480 cell viability after 72 h treatment with different concentrations of Myco (1, 2, 4, 8, 16 μg/mL) and 5-FU (1, 3, 9, 27, 81 μg/mL) for 72 **h. (D)** Results obtained after converting the mass units in Fig c to μM. The relative molecular mass of streptomycin is 1084.38, and that of 5-FU is 130.077.

Qualitative morphological assessment of SW480 cells was performed using light and scanning electron microscopy (SEM). Light microscopy revealed that control SW480 cells exhibited typical spindle-shaped morphology with pointed ends and a broader middle section. After 48 h of treatment with 8 μg/mL Myco, the cells shrank, displayed rough edges, and vacuoles appeared in the cytoplasm. Following treatment with 16 μg/mL Myco for the same duration, all cells transformed into a rounded shape, indicating significant cell death (Fig 2A). Scanning electron microscopy further demonstrated that the control group SW480 cells adhered tightly to the surface and maintained a regular spindle shape. In contrast, after Myco treatment, the number of apoptotic bodies and cell degradation products around the cells increased, and the degree of cell adhesion decreased. Notably, after treatment with 8 μg/mL Myco, the amount of debris surrounding the cells increased further, and the cell morphology became irregular (Fig 2B). The MTT assay was employed to assess the impact of Myco on the viability of SW480 cells, with results depicted in Figs 2C-D. Myco exhibited an inhibitory effect on cell proliferation in a dose-dependent manner, achieving a 100% inhibition rate at a concentration of 16 μg/mL. The IC50 values for Myco and 5-Fu against SW480 cells were determined to be 6.15 μg/ml (5.67 μM) and 3.05 μg/ml (23.46 μM), respectively.

### Myco blocks the SW480 cell cycle and induces apoptosis

**Fig 3:**
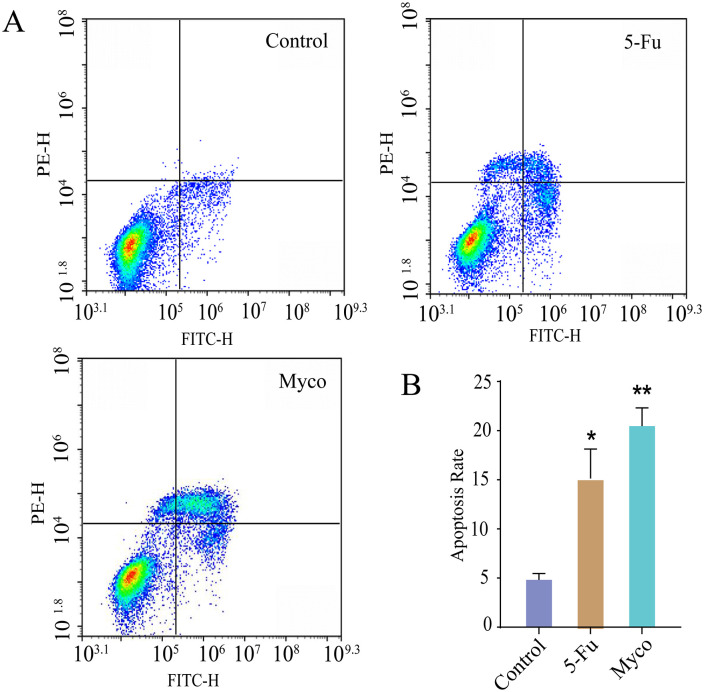
Effect of Myco on the cell cycle and apoptosis of SW480 cells. (A) the apoptosis rate of SW480 cells. (B) statistical data regarding the apoptosis rate. n = 3; data analyzed by one-way ANOVA; error bars represent mean ± SD.

Treatment with 8 µg/ml of Myco for 48 hours significantly increased the percentage of apoptotic cells in SW480 cells. The results indicated that 5-FU and Myco enhanced the proportion of apoptotic cells by 10.32% and 15.65%, respectively. This indicates that Myco significantly induces apoptosis in SW480 cells. Notably, the apoptotic effect of Myco was more pronounced compared to that of 5-FU (Figs 3A-B).

### Myco upregulates Bax and inhibits Bcl-2 expression in SW480 cells

**Fig 4:**
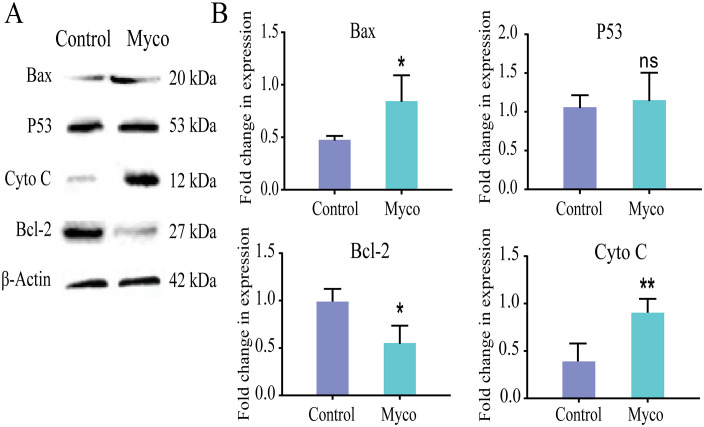
Effect of Myco on the levels of apoptosis-related proteins in SW480 cells. **(A)** Western blot analysis of apoptosis-related proteins in Myco-treated SW480 cells after 48 h, with β-actin as the internal reference protein. **(B)** Changes in the expression levels of the oncogene p53, the pro-apoptotic protein Bax, the anti-apoptotic protein Bcl-2, and Cyto C in SW480 cells. n = 3; data analyzed by one-way ANOVA; error bars represent mean ± SD.

To validate the molecular mechanism of Myco inhibition in SW480 cells predicted using network pharmacology, we examined changes in Bax and Bcl-2 pathway proteins following Myco treatment. Western blot analysis, revealed that Myco treatment did not significantly alter the expression level of the tumor suppressor gene p53 protein. However, Bax protein expression was increased by 1.88 ± 0.32-fold (*p* < 0.05), Bcl-2 protein expression was significantly decreased to 0.67 ± 0.11-fold of the control group(*p* < 0.05), and Cyto C expression was increased by 1.58 ± 0.17-fold (*p* < 0.01) (Fig 4).

### Myco promotes nuclear apoptosis and DNA damage in SW480 cells

**Fig 5:**
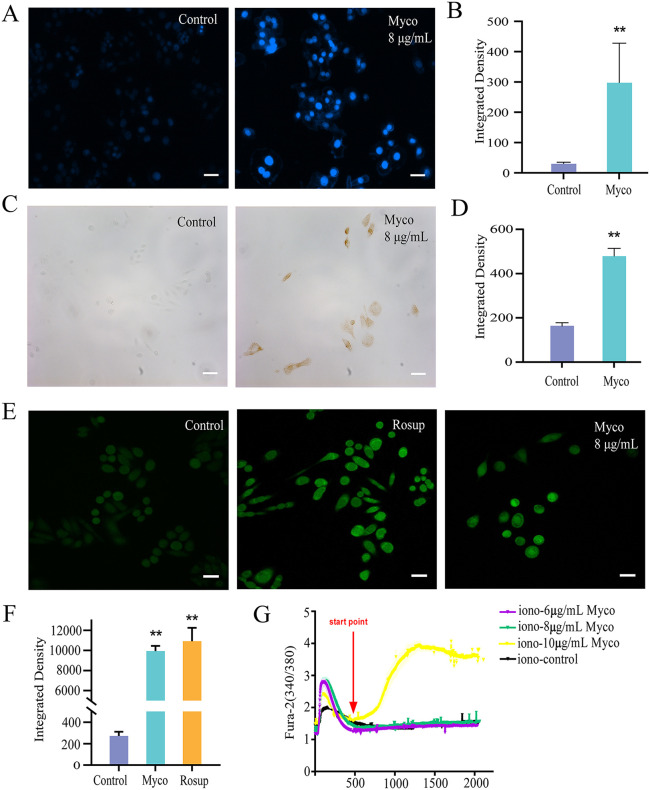
The impact of Myco on stress responses and homeostasis regulation in SW480 cells. **(A)** Hoechst 33342 staining method for detecting nuclear morphology of apoptosis in SW480 cells. **(B)** Fluorescence statistical graph of nuclei. **(C)** TUNEL method for detecting DNA damage in SW480 cells. **(D)** Fluorescence statistical graph of DNA damage. **(E)** ROS level in SW480 cells. **(F)** ROS fluorescence statistics. **(G)** Cytosolic calcium signaling level. Rosup: a positive control reagent used to induce intracellular ROS production. The scale bar is 50 μm. n = 3; data analyzed by one-way ANOVA; error bars represent mean ± SD.

To further explore the mechanism of Myco-induced apoptosis in SW480 cells, nuclear apoptosis, and DNA damage were observed. Myco treatment resulted in blue, densely stained nuclei and distinct apoptotic nuclear morphology(*p* < 0.01) (Figs 5A-B). Myco-treated SW480 cells exhibited reddish-brown staining after DAB coloring, indicating significant DNA breakage(*p* < 0.01) (Figs 5C-D). This suggests that the nuclear and DNA morphology of Myco-treated SW480 cells is consistent with the apoptotic phenotype. Meanwhile, this study examined the changes in intracellular ROS concentration and calcium ion concentration in SW480 cells treated with Myco, and found that compared to the control group, the green fluorescence intensity of Myco-treated SW480 cells increased by 3.53 ± 0.60-fold (*p* < 0.01) (Figs 5E-F). This finding indicates that Myco treatment elevates the intracellular ROS levels in SW480 cells. Results of intracellular calcium signaling assays showed that, compared with the control group, after depletion of intracellular calcium stores, the addition of calcium-buffered solutions containing 6 μg/mL or 8 μg/mL of Myco had no effect on intracellular calcium levels; however, when the concentration reached 10 μg/mL, intracellular calcium levels increased significantly 2.63 ± 0.09-fold (*p* < 0.01) (Fig 5G).

## Discussion

The development of low-toxicity and efficient natural lipopeptide compounds for the treatment of tumors and other diseases has good application prospects. In this study, the lipopeptide Myco was metabolized by *Bacillus subtilis* obtained by autonomous isolation [21]. SW480 colon cancer cells were used as the target to investigate the role of Myco in inhibiting colon cancer tumor proliferation and its possible regulatory mechanism.

Unlimited replication of tumor cells, metastasis, resistance to cell death, and genomic instability are characteristics of many cancers, including CRC [22]. Therefore, inducing apoptosis in tumor cells is an effective strategy for cancer treatment. Myco significantly reduced the viability of SW480 cells, exhibiting an IC50 value of 5.67 μM, which is notably lower than that of the commonly used colon cancer therapeutic drug, 5-FU. In comparison, Iturin A [23] and a surface activator [24], both of which belong to the same family of lipopeptides as Myco, demonstrated IC50 values of 30.89 μM and 26 μM, respectively, against colon cancer cells. This indicates that Myco has a stronger inhibitory effect on colon cancer cells, highlighting its greater potential for antitumor activity.

Observation of apoptosis-related phenotypes revealed that following Myco treatment, SW480 cells exhibited a wrinkled morphology, ragged edges, cytoplasmic vacuolization, and significant cell death. Myco may also act on the plasma membrane of target cells, thereby altering membrane permeability and generating ion-conducting pores [9]. Calcium ions are important second messengers in cells and are involved in multiple signaling pathways, including apoptosis [25]. Elevated calcium ion concentrations have been observed to potentially trigger apoptosis in cancer cells [26]. Therefore, by detecting changes in intracellular calcium ion concentrations, we sought to determine whether they were involved in Myco-induced apoptosis. The study found that low concentrations of Myco had no effect on intracellular calcium signaling levels, whereas at a concentration of 10 μg/mL, the intracellular calcium signaling levels increased significantly. This indicates a concentration-dependent threshold effect, and the higher concentration (10 μg/mL) may exceed the threshold for Myco-induced pore formation, thereby leading to a significant extracellular calcium influx. Combined with the results of the Annexin V-FITC/PI double-staining assay for apoptosis, Myco at 8 μg/mL significantly induced apoptosis in SW480 cells. This suggests that intracellular calcium ions are not sensitive to Myco-induced apoptosis; however, higher concentrations of Myco may alter cell membrane permeability, thereby leading to a significant increase in intracellular calcium ion concentration. This is consistent with the membrane-active properties of lipopeptides [27,28]. Furthermore, Myco treatment increased ROS levels in SW480 cells. Although ROS are byproducts of normal metabolism or detrimental exogenous exposure, they play a crucial role in cellular damage during activation and are key regulators of apoptosis [29,30].

Analysis of apoptotic protein expression revealed that Myco treatment significantly downregulated Bcl-2 expression in SW480 cells. The regulatory mechanisms underlying this phenomenon are likely to be multifaceted and complex. Molecular docking analysis revealed that Myco binds to Bcl-2 protein, suggesting a potential direct interaction between the two and providing important clues for further mechanistic investigation. However, the binding of a protein to its ligand does not directly lead to the degradation of the target protein. Studies have shown that differences in the anti-apoptotic capacity among Bcl-2 family members primarily depend on their protein stability, with less stable members (such as Mcl-1 and Bfl-1) being prone to steady-state or drug-induced proteasomal degradation [3,31]. We hypothesized that the Myco-induced reduction in Bcl-2 protein levels may result from Myco-induced conformational changes in the Bcl-2 protein, thereby facilitating its degradation. Previous studies have found that active compounds from *Bacillus subtilis* primarily induce endogenous apoptotic pathways; the mechanism of action of Myco substances may be similar. Following treatment, they induced the expression of the pro-apoptotic protein Bax and inhibited the expression of the anti-apoptotic protein Bcl-2, leading to apoptosis. The Bcl-2 family further regulates the release of apoptotic factors, such as cytochrome C, from the mitochondria, thereby inducing endogenous apoptosis [32,33]. Although our data confirm that Myco downregulates Bcl-2 protein expression and can bind directly to Bcl-2 in computer simulations, further studies are required to elucidate the precise mechanism by which Myco interacts with Bcl-2 in vivo. Future studies should assess the ubiquitination status of Bcl-2, investigate the activation of JNK and its role in Myco-induced Bcl-2 downregulation, and explore whether proteasome inhibitors can reverse the reduction in Bcl-2 levels. Such mechanistic studies are crucial for elucidating the full therapeutic potential of Myco as an anti-cancer drug.

The limitation of this study is that only the inhibitory effect of Myco on colon cancer SW480 cells was investigated in vitro, and the inhibitory effect in vivo has not been further studied. Subsequent research needs to comprehensively consider various factors in an in vivo tumor model, such as drug metabolism, interactions with the immune system, and the influence of different tissues and organs. This will allow for a more accurate evaluation of the inhibitory effect of Myco on colon cancer in CRC in vivo and its potential application in CRC treatment of colon cancer, thereby providing a more solid theoretical foundation and data support for the clinical application and translation of these research findings.

The findings of this study suggest that Myco may play a significant role in apoptosis by targeting the Bcl-2/Bax pathway. Additionally, it appears to inhibit the development of colon tumors through various mechanisms, including the suppression of cell proliferation, induction of apoptosis, and enhancement of intracellular ROS production. This study provides a theoretical foundation for the clinical application of Myco in colon cancer treatment.

## Conclusion

The findings of this study suggest that Myco may play a significant role in the process of apoptosis by targeting the Bcl-2/Bax pathway. Furthermore, it appears to inhibit the development of colon tumors through various mechanisms, including the suppression of cell proliferation, induction of apoptosis, and enhancement of intracellular ROS production. This study provides a theoretical foundation for the clinical application of Myco in colon cancer treatment, holding potential for the development of a new generation of anti-cancer drugs.

## Supporting information

S1 Fig Myco’s HPLC separation chromatograms and structural diagrams. (A) separation chromatogram. (B) structural diagram.
(PDF)

S1 Table Protein targets, accession numbers, and PDB structures used for molecular docking analysis. (PDF)

## References

[1] Siegel RL, Wagle NS, Cercek A, Smith RA, Jemal A. Colorectal cancer statistics, 2023. CA Cancer J Clin. 2023;73:233–54. doi: 10.3322/caac.21772 36856579

[2] Madukwe JC. Overcoming drug resistance in cancer. Cell. 2023;186(8):1515–6. doi: 10.1016/j.cell.2023.03.019 37059057

[3] Basu A. The interplay between apoptosis and cellular senescence: Bcl-2 family proteins as targets for cancer therapy. Pharmacol Ther. 2022;230:107943. doi: 10.1016/j.pharmthera.2021.107943 34182005

[4] Gitego N, Agianian B, Mak OW, Kumar Mv V, Cheng EH, Gavathiotis E. Chemical modulation of cytosolic BAX homodimer potentiates BAX activation and apoptosis. Nat Commun. 2023;14(1):8381. doi: 10.1038/s41467-023-44084-3 38104127 PMC10725471

[5] Goel S, Bergholz JS, Zhao JJ. Targeting CDK4 and CDK6 in cancer. Nat Rev Cancer. 2022;22(6):356–72. doi: 10.1038/s41568-022-00456-3 35304604 PMC9149100

[6] Ding M, Dong C, Mao Y, Liu S, Zhao Y, Wang X. A combined network pharmacology and molecular biology approach to investigate the potential mechanisms of G-M6 on ovarian cancer. Bioorg Chem. 2023;138:106657. doi: 10.1016/j.bioorg.2023.106657 37302316

[7] Hu S, Li S, Xu Y, Huang X, Mai Z, Chen Y, et al. The antitumor effects of herbal medicine Triphala on oral cancer by inactivating PI3K/Akt signaling pathway: based on the network pharmacology, molecular docking, in vitro and in vivo experimental validation. Phytomedicine. 2024;128:155488. doi: 10.1016/j.phymed.2024.155488 38493718

[8] Nimbeshaho F, Nihorimbere G, Arias AA, Liénard C, Steels S, Nibasumba A, et al. Unravelling the secondary metabolome and biocontrol potential of the recently described species Bacillus nakamurai. Microbiol Res. 2024;288:127841. doi: 10.1016/j.micres.2024.127841 39153465

[9] Liu Q, Wang L, He D, Wu Y, Liu X, Yang Y, et al. Application Value of Antimicrobial Peptides in Gastrointestinal Tumors. Int J Mol Sci. 2023;24(23):16718. doi: 10.3390/ijms242316718 38069041 PMC10706433

[10] Liu X, Tao X, Zou A, Yang S, Zhang L, Mu B. Effect of the microbial lipopeptide on tumor cell lines: apoptosis induced by disturbing the fatty acid composition of cell membrane. Protein Cell. 2010;1(6):584–94. doi: 10.1007/s13238-010-0072-4 21204010 PMC4875320

[11] Leclère V, Béchet M, Adam A, Guez J-S, Wathelet B, Ongena M, et al. Mycosubtilin overproduction by Bacillus subtilis BBG100 enhances the organism’s antagonistic and biocontrol activities. Appl Environ Microbiol. 2005;71(8):4577–84. doi: 10.1128/AEM.71.8.4577-4584.2005 16085851 PMC1183317

[12] Morejón MC, Laub A, Kaluđerović GN, Puentes AR, Hmedat AN, Otero-González AJ, et al. A multicomponent macrocyclization strategy to natural product-like cyclic lipopeptides: synthesis and anticancer evaluation of surfactin and mycosubtilin analogues. Org Biomol Chem. 2017;15(17):3628–37. doi: 10.1039/c7ob00459a 28406518

[13] Zhao H, Yan L, Xu X, Jiang C, Shi J, Zhang Y, et al. Potential of Bacillus subtilis lipopeptides in anti-cancer I: induction of apoptosis and paraptosis and inhibition of autophagy in K562 cells. AMB Express. 2018;8(1):78. doi: 10.1186/s13568-018-0606-3 29777449 PMC5959823

[14] Hajare SN, Subramanian M, Gautam S, Sharma A. Induction of apoptosis in human cancer cells by a Bacillus lipopeptide bacillomycin D. Biochimie. 2013;95(9):1722–31. doi: 10.1016/j.biochi.2013.05.015 23770444

[15] Dey G, Bharti R, Dhanarajan G, Das S, Dey KK, Kumar BNP, et al. Marine lipopeptide Iturin A inhibits Akt mediated GSK3β and FoxO3a signaling and triggers apoptosis in breast cancer. Sci Rep. 2015;5:10316. doi: 10.1038/srep10316 25974307 PMC4431395

[16] Dey G, Bharti R, Ojha PK, Pal I, Rajesh Y, Banerjee I, et al. Therapeutic implication of “Iturin A” for targeting MD-2/TLR4 complex to overcome angiogenesis and invasion. Cell Signal. 2017;35:24–36. doi: 10.1016/j.cellsig.2017.03.017 28347875

[17] Fickers P, Leclère V, Guez J-S, Béchet M, Coucheney F, Joris B, et al. Temperature dependence of mycosubtilin homologue production in Bacillus subtilis ATCC6633. Res Microbiol. 2008;159(6):449–57. doi: 10.1016/j.resmic.2008.05.004 18656330

[18] Aimaier R, Li H, Cao W, Cao X, Zhang H, You J, et al. The Secondary Metabolites of Bacillus subtilis Strain Z15 Induce Apoptosis in Hepatocellular Carcinoma Cells. Probiotics Antimicrob Proteins. 2025;17(2):832–42. doi: 10.1007/s12602-023-10181-4 37906413

[19] Li H, Zhou D, Wang W, Aimaier R, Jun Y, Zhao H, et al. Mycosubtilin Induces G1 Phase Block and Autophagy in Cervical Cancer HeLa Cells. Probiotics Antimicrob Proteins. 2026;18(1):457–69. doi: 10.1007/s12602-025-10534-1 40240746

[20] Cao X-Y, Aimaier R, Yang J, Yang J, Chen Z-Y, Zhao J-J, et al. Effect of bacillus subtilis strain Z15 secondary metabolites on immune function in mice. BMC Genomics. 2023;24(1):273. doi: 10.1186/s12864-023-09313-5 37208602 PMC10198031

[21] Lin R, Zhang Q, Yin L, Zhang Y, Yang Q, Liu K, et al. Isolation and characterization of a mycosubtilin homologue antagonizing Verticillium dahliae produced by Bacillus subtilis strain Z15. PLoS One. 2022;17(6):e0269861. doi: 10.1371/journal.pone.0269861 35696380 PMC9191732

[22] Wang Z, Tang X, Wu X, Yang M, Wang W, Wang L, et al. Cardamonin exerts anti-gastric cancer activity via inhibiting LncRNA-PVT1-STAT3 axis. Biosci Rep. 2019;39(5):BSR20190357. doi: 10.1042/BSR20190357 31028131 PMC6522749

[23] Zhao H, Xu X, Lei S, Shao D, Jiang C, Shi J, et al. Iturin A-like lipopeptides from Bacillus subtilis trigger apoptosis, paraptosis, and autophagy in Caco-2 cells. J Cell Physiol. 2019;234(5):6414–27. doi: 10.1002/jcp.27377 30238995

[24] Kim S-Y, Kim JY, Kim S-H, Bae HJ, Yi H, Yoon SH, et al. Surfactin from Bacillus subtilis displays anti-proliferative effect via apoptosis induction, cell cycle arrest and survival signaling suppression. FEBS Lett. 2007;581(5):865–71. doi: 10.1016/j.febslet.2007.01.059 17292358

[25] Fang S, Wang Y, Huang Z, Huang Z. STIM1-mediated calcium signalling in cancer: Its relation to tumour aggressiveness and therapeutic horizons. Biochim Biophys Acta Rev Cancer. 2025;1880(5):189420. doi: 10.1016/j.bbcan.2025.189420 40816544

[26] Ren T, Tang Y-J, Wang M-F, Wang H-S, Liu Y, Qian X, et al. Triptolide induces apoptosis through the calcium/calmodulin‑dependent protein kinase kinaseβ/AMP‑activated protein kinase signaling pathway in non‑small cell lung cancer cells. Oncol Rep. 2020;44(5):2288–96. doi: 10.3892/or.2020.7763 33000264

[27] Xie Y, Peng Q, Ji Y, Xie A, Yang L, Mu S, et al. Isolation and Identification of Antibacterial Bioactive Compounds From Bacillus megaterium L2. Front Microbiol. 2021;12:645484. doi: 10.3389/fmicb.2021.645484 33841370 PMC8024468

[28] Lim AL, Miller BW, Fisher MA, Haygood MG, Barrows LR, Schmidt EW. Differential membrane lipid disruption by lipopeptide antibiotics, colistin and turnercyclamycins. Nat Commun. 2026;17(1):1880. doi: 10.1038/s41467-026-68681-0 41554749 PMC12923534

[29] Liu L, Yang C, Zhu L, Wang Y, Zheng F, Liang L, et al. RSL3 enhances ROS-mediated cell apoptosis of myelodysplastic syndrome cells through MYB/Bcl-2 signaling pathway. Cell Death Dis. 2024;15(7):465. doi: 10.1038/s41419-024-06866-5 38956026 PMC11219730

[30] Moloney JN, Cotter TG. ROS signalling in the biology of cancer. Semin Cell Dev Biol. 2018;80:50–64. doi: 10.1016/j.semcdb.2017.05.023 28587975

[31] Ashkenazi A, Fairbrother WJ, Leverson JD, Souers AJ. From basic apoptosis discoveries to advanced selective BCL-2 family inhibitors. Nat Rev Drug Discov. 2017;16(4):273–84. doi: 10.1038/nrd.2016.253 28209992

[32] Morse PT, Pérez-Mejías G, Wan J, Turner AA, Márquez I, Kalpage HA, et al. Cytochrome c lysine acetylation regulates cellular respiration and cell death in ischemic skeletal muscle. Nat Commun. 2023;14(1):4166. doi: 10.1038/s41467-023-39820-8 37443314 PMC10345088

[33] Shajahan AN, Dobbin ZC, Hickman FE, Dakshanamurthy S, Clarke R. Tyrosine-phosphorylated caveolin-1 (Tyr-14) increases sensitivity to paclitaxel by inhibiting BCL2 and BCLxL proteins via c-Jun N-terminal kinase (JNK). J Biol Chem. 2012;287(21):17682–92. doi: 10.1074/jbc.M111.304022 22433870 PMC3366801

