# Supplementary material for: Inhibitory effects of mycosubtilin on proliferation of colon cancer SW480 cells
Source: PLoS One. 2026 May 20;21(5):e0348056. doi: 10.1371/journal.pone.0348056 (PMC13189412; doi:10.1371/journal.pone.0348056)
Supplement: S1 Fig — (A) separation chromatogram. (B) structural diagram. (PDF) [file pone.0348056.s001.pdf]

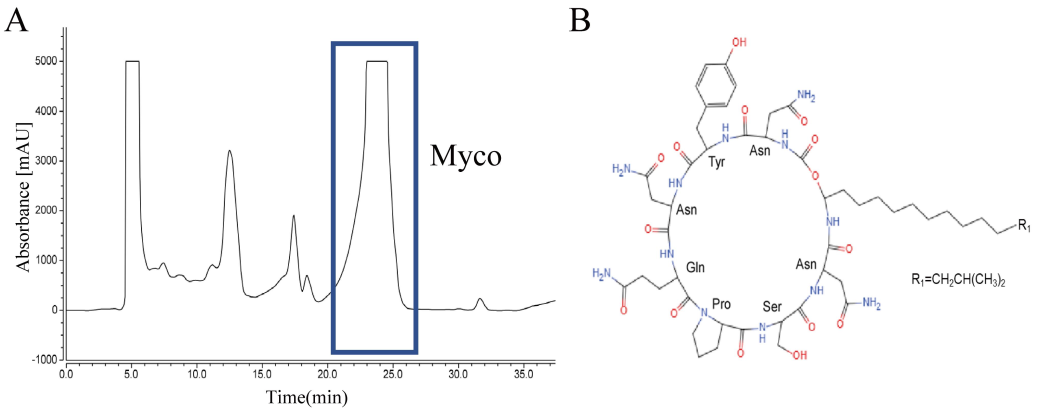

S1 Fig. Myco's HPLC separation chromatograms and structural diagrams. (A) separation chromatogram. (B) structural diagram.
