# Supplementary material for: Inhibitory effects of mycosubtilin on proliferation of colon cancer SW480 cells
Source: PLoS One. 2026 May 20;21(5):e0348056. doi: 10.1371/journal.pone.0348056 (PMC13189412; doi:10.1371/journal.pone.0348056)
Supplement: S1 Table — (PDF) [file pone.0348056.s002.pdf]

S1 Table. Protein targets, accession numbers, and PDB structures used for molecular docking analysis.

Protein	UniProt ID	PDB ID	Resolution (Å)
HIF1A	Q16665	1H2K	2.15 Å
STAT3	P40763	5AX3	2.98 Å
CASP3	P42574	1CP3	2.30 Å
AKT1	P31749	1H10	1.40 Å
BCL2	P10415	2W3L	2.10 Å
SRC	P12931	1A07	2.20 Å
GSK3B	P49841	1GNG	2.60 Å
STAT1	P42224	1BF5	2.90 Å
CASP8	Q14790	1F9E	2.90 Å
HSP90AA1	P07900	1BYQ	1.50 Å
PPARG	P37231	1FM6	2.10 Å
PARP1	P09874	1UK0	3.00 Å
MDM2	Q00987	1RV1	2.30 Å
ESR1	P03372	1A52	2.80 Å
BCL2L1	Q07817	1MAZ	2.20 Å
ALB	P02768	1AO6	2.50 Å
JAK2	O60674	2B7A	2.00 Å
ANXA5	P08758	1ANW	2.40 Å
HSP90AB1	P08238	1QZ2	3.00 Å
NFKB1	P19838	1NFI	2.70 Å
MMP9	P14780	1GKC	2.30 Å
MAP2K1	Q02750	1S9J	2.40 Å
EGFR	P00533	1IVO	3.30 Å
MMP2	P08253	1CK7	2.80 Å
IGF1R	P08069	1IGR	2.60 Å
